# Hierarchical Data-Driven Analysis of Clinical Symptoms Among Patients With Parkinson's Disease

**DOI:** 10.3389/fneur.2019.00531

**Published:** 2019-05-21

**Authors:** Tal Kozlovski, Alexis Mitelpunkt, Avner Thaler, Tanya Gurevich, Avi Orr-Urtreger, Mali Gana-Weisz, Netta Shachar, Tal Galili, Mira Marcus-Kalish, Susan Bressman, Karen Marder, Nir Giladi, Yoav Benjamini, Anat Mirelman

**Affiliations:** ^1^Department of Statistics and Operations Research, Tel Aviv University, Tel Aviv, Israel; ^2^Sackler School of Medicine, Tel Aviv University, Tel Aviv, Israel; ^3^Pediatric Neurology Unit, Dana Children Hospital, Tel Aviv Medical Center, Tel Aviv, Israel; ^4^Movement Disorders Unit, Tel Aviv Medical Center, Neurological Institute, Tel Aviv, Israel; ^5^Sagol School of Neuroscience, Tel Aviv University, Tel Aviv, Israel; ^6^Genetic Institute, Tel Aviv Medical Center, Tel Aviv, Israel; ^7^Department of Neurology, Icahn School of Medicine at Mount Sinai, Mount Sinai Beth Israel Medical Center, New York, NY, United States; ^8^Department of Neurology, Taub Institute for Alzheimer's Disease and the Aging Brain, College of Physicians and Surgeons, Columbia University, New York, NY, United States; ^9^Edmond J. Safra Center for Bioinformatics, Tel Aviv University, Tel Aviv, Israel; ^10^Laboratory of Early Markers of Neurodegeneration, Tel Aviv Medical Center, Neurological Institute, Tel Aviv, Israel

**Keywords:** Parkinson's disease, G2019S-LRRK2, GBA, hierarchical testing, selective inference

## Abstract

Mutations in the LRRK2 and GBA genes are the most common inherited causes of Parkinson's disease (PD). Studies exploring phenotypic differences based on genetic status used hypothesis-driven data-gathering and statistical-analyses focusing on specific symptoms, which may influence the validity of the results. We aimed to explore phenotypic expression in idiopathic PD (iPD) patients, G2019S-LRRK2-PD, and GBA-PD using a data-driven approach, allowing screening of large numbers of features while controlling selection bias. Data was collected from 1525 Ashkenazi Jews diagnosed with PD from the Tel-Aviv Medical center; 161 G2019S-LRRK2-PD, 222 GBA-PD, and 1142 iPD (no G2019S-LRRK2 or any of the 7 AJ GBA mutations tested). Data included 771 measures: demographics, cognitive, physical and neurological functions, performance-based measures, and non-motor symptoms. The association of the genotypes with each of the measures was tested while accounting for age at motor symptoms onset, gender, and disease duration; *p*-values were reported and corrected in a hierarchical approach for an average over the selected measures false discovery rate control, resulting in 32 measures. GBA-PD presented with more severe symptoms expression while LRRK2-PD had more benign symptoms compared to iPD. GBA-PD presented greater cognitive and autonomic involvement, more frequent hyposmia and REM sleep behavior symptoms while these were less frequent among LRRK2-PD compared to iPD. Using a data-driven analytical approach strengthens earlier studies and extends them to portray a possible unique disease phenotype based on genotype among AJ PD. Such findings could help direct a more personalized therapeutic approach.

## Introduction

Mutations in the LRRK2 and GBA genes are the most common known genetic risk factors of Parkinson's disease (PD) (1, 2). The phenotype of genetic-associated PD has been described mainly compared to idiopathic PD (iPD). Some reported similarities in disease symptoms between LRRK2-PD and iPD (3), while others found a higher frequency of the postural instability gait difficulty subtype (4, 5), with less non-motor symptoms (4, 6–10) in LRRK2-PD. GBA-PD phenotype points to a younger age of motor symptoms onset, earlier and higher rate of cognitive decline and faster rate of progression compared with iPD (11–14).

Establishing differences between genetic-associated PD and iPD may help to understand the molecular pathogenesis of the disease and ultimately lead to new therapeutic strategies. However, studies comparing phenotype in the three groups using identical methods are lacking. In addition, previous explorations were based on a hypothesis driven approach, comparing specific features or data summaries between groups, and adjusting (in best case scenarios) for a limited number of multiple comparisons. The magnitude of variables measured and the breadth of domains are often large. Such abundance of data requires accounting for multiple comparisons and selective inference in order to maintain results replicability (15, 16), and avoid loss of information because of summation into means and total scores. Data-driven analysis enables the inclusion of large numbers of measures while controlling for False Discovery Rate (FDR), both for dimensions reduction and for hypotheses testing. The aim of this study was to explore phenotypic expression in iPD, LRRK2-PD and GBA-PD using a well-guarded data-driven approach.

## Methods

### Participants and Procedures

The study was conducted in the Movement Disorder Unit at the Tel-Aviv Medical Center between 2005 and 2015. Patients were included in the study if they were of Ashkenazi Jewish (AJ) descent, and fulfilled the UK PD Brain bank criteria (including patients with family history) (17). All AJ PD patients who approached any of the neurologists in the MDU (tertiary center in Tel-Aviv) were offered to participate in this observational study. The study was approved by the ethical committee of the Tel Aviv Medical Center. All patients signed an informed written consent prior to participation.

Upon inclusion, 1525 patients were screened for the seven most common AJ GBA mutations (N370S, L444P, c.84insG, IVS2+1G->A, V394L, R496H, and 370Rec) and the G2019S mutation in the LRRK2 gene. Patients underwent a wide battery of medical exams and questionnaires assessing motor and non-motor symptoms (Table 1). Information on disease symptoms and management were collected from structured interviews and medical charts as well as using standardized questionnaires before the genetic status of each patient was ascertained (2, 4, 10, 18). Disease severity was assessed using the Unified Parkinson Disease Rating Scale (UPDRS part III) (19) and the H&Y staging (20). Cognitive function was evaluated using the Montreal Cognitive Assessment test (MoCA) (21), Stroop test (22), verbal fluency (23), and Trail Making Test (TMT color version) (24). Depression was assessed using the Beck Depression Inventory (BDI) (25) and the Geriatric Depression Scale (GDS) (26), anxiety was measured using the Spielberger State and Trait Anxiety Inventory (27). The Non-Motor Symptom Questionnaire (NMS) (28) and the Scale for Autonomic Function (SCOPA-AUT) (29) were used to assess autonomic function. Olfaction was assessed using the University of Pennsylvania Smell Identification Test (UPSIT) (21), hyposmia was defined based on age and gender cut-offs and the REM sleep Behavior Questionnaire (RBDQ) (30) was administered to evaluate RBD. Patients were assessed during morning office hours and were requested not to alter their medication schedule, thus tested during “ON” medication condition.

**Table 1:** Characteristics of study participants.

**Variable**	***GBA***	***LRRK2***	**Non carriers**	**Total observations**
Number	222	161	1,142	1,525
Age at onset, years (*SD*) [range in years]	58.3 (11.08) [24-85]	58.1 (10.97) [28-91]	60.9 (11.39) [20-94]	1,525
Age at enrollment, years (*SD*) [range in years]	65.5 (10.5) [33-89]	66.5 (10.11) [36-93]	67.6 (10.52) [29-96]	1,525
**GENDER**				
Male *n* (%)	129 (58.11)	82 (50.93)	734 (64.27)	1,525
Male/Female ratio	1.39	1.04	1.8	
**FAMILY HISTORY OF PD**				
1st degree relative with PD *n* (%)	44 (19.82%)	51 (31.67%)	192 (16.81%)	1,489
Total with any family history of PD *n* (%)	81 (36.48%)	80 (49.69%)	306 (26.79%)	1,496
**BMI**				
mean (*SD*)	26.6 (5.23)	26.1 (3.77)	26.6 (5.38)	462

### Standard Protocol Approvals, Registrations, and Patient Consents

The study was approved by the local ethical committee at Tel Aviv Medical Center and was performed according to the principles of the Declaration of Helsinki. Eligible participants provided informed written consent.

## Statistical Analysis

**Figure 1:**
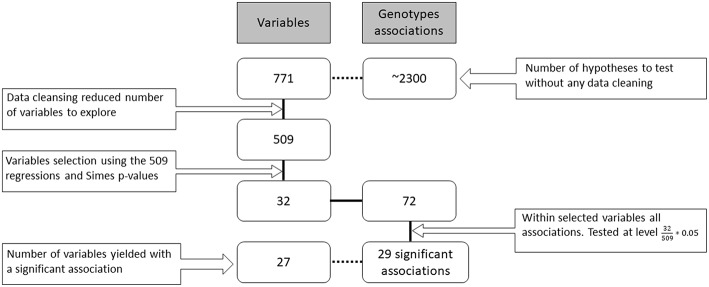
Research workflow. Data gathering, cleansing, screening, hypotheses testing and number of findings. The boxes on the left side of the figure indicate the number of variables (families of hypotheses) in each part of the analysis. On the right side are the total number of genotype associations considered for each of the family hypotheses (individual hypotheses). The solid black connecting line represents the actual workflow of anlaysis while the dashed connecting line describes additional information on the data. The number of final family variables in the analysis was 32 for which 72 hypotheses were examined resulting in 29 significant hypotheses.

Overall, 771 potential measures were available about the patients in various databases (resulting flatly in ~2300 genotype associations to be screened). They included both fine and gross level measures, for example, both the individual questions in a questionnaire and their overall scores. Each of the 1,525 participants contributed a different subset of those measures (due to time limits, availability of questionnaires, etc.) during their visits to the research center. In the data cleansing stage, dozens of variables were removed due to redundancy of information or if more than 99% of records were missing. The remaining 509 measures were tested for their association with the genotypes in the following hierarchical way (see Figure 1).

Each measure was fitted a generalized linear model with the genotype as a categorical explanatory variable, where iPD patients were considered as the reference level. The model further included 3 covariates: age at onset, disease duration and gender, in order to adjust for their potential influence. The generalized model used was either linear, logistic, ordered logistic or multinomial, according to the measure's type. Furthermore, if the contingency table of genotype and the categorical measure had at least one cell with <2 observations we used a logistic regression with bias reduction (31, 32).

**Figure 2:**
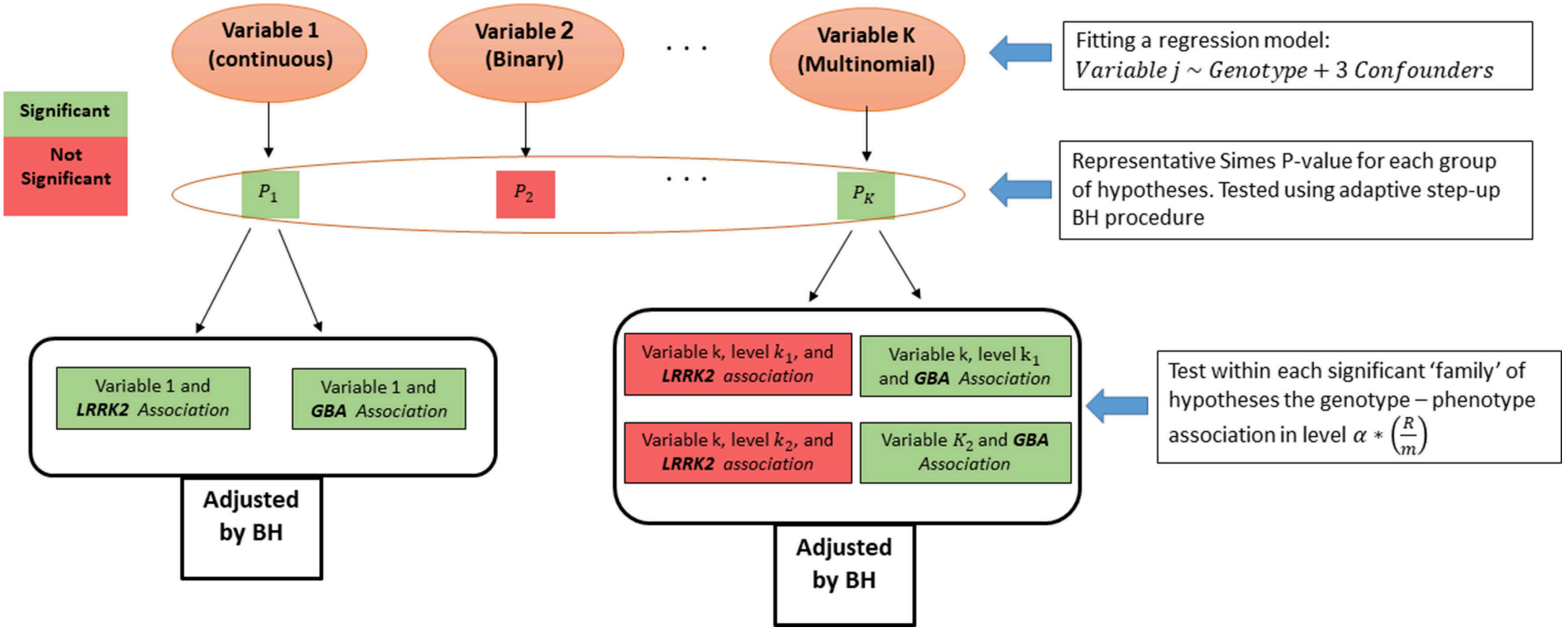
Statistical analysis algorithm. Measure screening and hypotheses testing algorithm.

From each regression model we obtained p-values for the two genotypes effects, and for the categorical variables we obtained a pair for each one of the categories of the dependent measure. Associations related to the same measure were grouped into a family of hypotheses, and their intersection is the hypothesis of no association of the measure with any genotype. As suggested before (16), we first tested the above intersection hypothesis across the measures. For that, a p-value for each intersection hypothesis was calculated using Simes test (33). Then, the adaptive step-up Benjamini-Hochberg (BH) procedure (34) was used to select the measures that have some significant association with any of the genotypes (35), while guaranteeing FDR control on the selected measures. Finally, after the measures screening stage, the family of associations related to each selected measure were tested again using the FDR control procedure. The level of the test at the second stage was set according to Benjamini and Bogomolov (16) to control the average FDR over the selected groups (see Figure 2).

After adjusting the *p*-values with the BH procedure within each group, the threshold for significance within the selected groups was reduced from α by the proportion of the number of selected measures (*R*) out of total tested (*m*) $q=\left(\frac{R}{m}\right)*\;\alpha$ (in this case: $\left(\frac{32}{509}\right)*\;\alpha$). All the presented p-values below were adjusted to keep the average error rate over the selected groups, are denoted as *p*^*adj*^, and hence can be compared to the regular 0.05 and 0.1 thresholds. Analysis was preformed using R software version 3.2.4.

## Results

**Figure 3:**
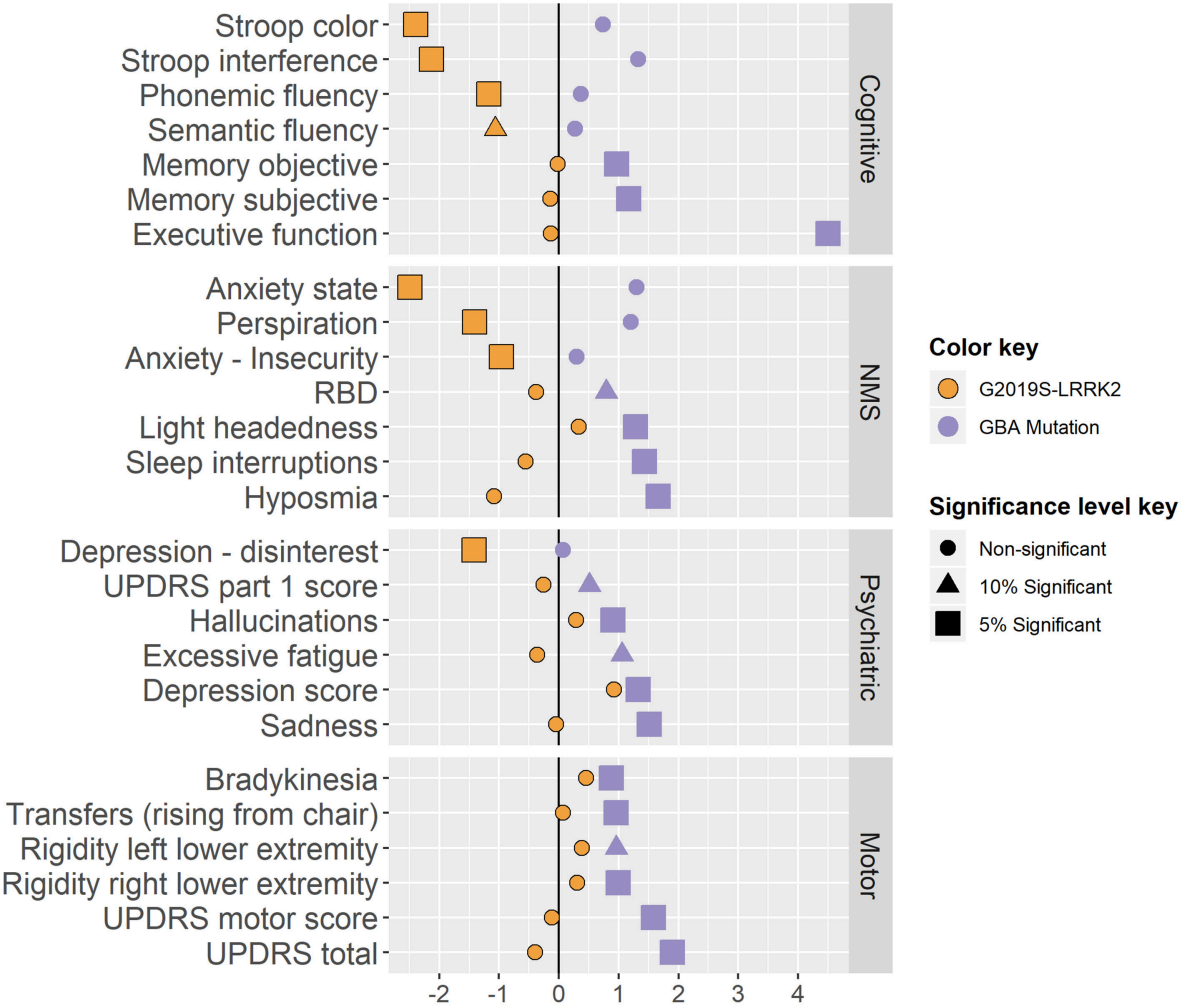
Results standardized effect sizes. Standardized effect sizes divided by domains and phenotypes of the two genotypes coefficients. The calculation of the standardized effect sizes varies across the different linear models as follows:
• Linear regression models: $\frac{\beta_{genotype}}{SD\left(y\;\right)}$• Logistic regression model (multinomial and binomial models): log(*OR*)• Ordered logistic regression models: log(*Cumulative OR*) The effect sizes are colored by genotype, and their shape and size present the minimal significance level they pass (0.05, 0.1 or none of them).

A total of 1525 AJ patients with PD participated in this study: 1142 iPDs, 161 carriers of the G2019S-LRRK2 mutation and 222 carriers of the 7 common AJ mutations in the GBA gene. The majority of the mutations in the GBA gene (65%) were considered mild GBA (N370S and R496H). Due to the small proportion of the other mutations, all carriers of the GBA gene were considered one group. In the second stage 72 associations were tested, 36 for each of the mutations. Twelve associations of LRRK2-PD and 17 associations of the GBA-PD were significantly different from the iPDs at the 0.05 level. Five more associations were found significant at a 0.1 level. Below we detail the specific differences in phenotype. The respective effect sizes are presented in Figure 3.

### Cognition

LRRK2-PD performed better than iPD patients on cognitive tests such as the congruent Stroop, Stroop interference and Verbal fluency tests (*p*^*adj*^ are < 0.001, 0.0047, 0.056 & 0.039 respectively). GBA–PD showed more difficulty in executive function (TMT test A-B; *p*^*adj*^ = 0.002) and reported more subjective memory complaints (*p*^*adj*^ = 0.017) and concentration difficulties (*p*^*adj*^ = 0.016). The MoCA test was not sensitive enough to pass the screening stage (Simes *p*^*adj*^ = 0.127).

### Non-motor Symptoms

GBA-PD were more hyposmic (*p*^*adj*^ = 0.009) and had more complains about headaches (*p*^*adj*^ < 0.001) compared to patients with iPD. GBA-PD had higher scores on the RBDQ (*p*^*adj*^ = 0.057), and more specifically reported more frequent awakenings during nighttime sleep (*p*^*adj*^ = 0.008). LRRK2-PD were correlated with less reports of perspiration (*p*^*adj*^ = 0.001).

### Mood and Psychiatric Symptoms

LRRK2-PD reported less activity withdrawal (GDS question 2) and scored lower on both the State and Trait Anxiety Inventory (*p*^*adj*^ are 0.006 and 0.045 respectively), while GBA-PD reported less satisfaction from their lives (*p*^*adj*^ = 0.012), and more symptoms of depression (from the BDI, *p*^*adj*^ are 0.01 and 0.041).

In addition, a larger percentage of GBA-PD reported hallucinations (NMSQ question 14, *p*^*adj*^ = 0.052) and in total received higher total scores in the UPDRS-part I (*p*^*adj*^ = 0.057).

### Motor Symptoms

GBA-PD presented with greater motor signs on the UPDRS-part III compared to iPD patients (*p*^*adj*^ = 0.022). More specifically, GBA-PD presented with more bradykinesia, difficulty in transfers and rigidity (questions 23.b and 27, *p*^*adj*^ are 0.003 and 0.023 and question 22.d and 22.e, *p*^*adj*^ are < 0.001 and 0.057 , respectively) compared to iPD and had a higher total UPDRS score (*p*^*adj*^ = 0.019).

### Other Measures

Six additional measures that passed the screening stage (AJ origin, number of children, initiation of dopa-Y/N, work hazard-Y/N, maternal mother risk-Y/N, and a clinically duplicate score from the GDS questionnaire) were of no clinical implications and are therefore omitted from Figure 3. Omitting the duplicated variable made no change in the results of the analysis; when omitting all six from the analysis, the FDR at the families' level increased to 0.069 (see Supplementary Material for a detailed version of Figure 3).

## Discussion

This paper presents findings based on the exploration of a large set of data in order to assess phenotype-genotype associations in PD. We used a new hierarchical statistical testing approach that facilitates analysis of a large sets of data from different domains, incorporates all data points without summation of tests with the ability to explore directionality without prior hypothesis. Furthermore, the methodology allows inference on the results while controlling an appropriate error rate, thus ensuring the validity of the discoveries. Our exploration provided evidence of differences in phenotypes based on genetic mark up (Figure 3). The results validate through replication some previous results in the literature, as well as refuting others.

Differences between iPD, LRRK2-PD, and GBA-PD were found in multiple domains. In general, despite adjusting for disease duration, gender and age at onset, patients with GBA-PD presented with more severe symptoms and signs while LRRK2-PD had more moderate symptoms and signs as compared to iPD. Consistent with previous studies, our analysis showed greater cognitive impairment in patients with GBA-PD than iPD (2, 36–39), while LRRK2-PD demonstrated more preserved cognitive functions. Patients with GBA-PD also showed more autonomic involvement, more hyposmia and RBD symptoms, while olfaction disturbances and RBD were minimal in LRRK2-PD as compared to iPD. This is consistent with previous studies reporting on greater autonomic involvement in GBA related PD (11, 37) and less hyposmia and RBD in LRRK2-PD (9, 40–42).

Reports on depression, anxiety and psychiatric involvement in LRRK2 related PD are equivocal in the literature. Marras et al. reported higher BDI scores in patients with LRRK2 associated PD (3), while Ben-Sassi et al. reported less depressive symptoms in a cohort of Tunisians with G2019S-LRRK2 (43, 44). Our findings support the latter, with LRRK2-PD patients showing significantly lower apathy and hallucinations than iPD, and presenting with less depressive and anxiety symptoms than iPD, and less psychiatric involvement compared to GBA-PD.

Previously we and others reported that LRRK2 related PD was more frequently associated with postural instability and gait difficulty phenotype and presented with more motor involvement (4, 5). In this study, we observed greater motor involvement in the GBA-PD group than the LRRK2 related PD while the LRRK2-PD had more motor involvement than iPD (recall Figure 3). This finding is interesting and potentially relates to the methodology of assessment. In this study, the assessment of motor function was based on the UPDRS and did not include sensitive quantifiable gait assessment such as in our previous work (5). Such quantifiable assessment could provide additional information that is not detected in the UPDRS. This may also reflect the vigor of data-driven analysis and the strength of including multiple domains in the search, while controlling for selection stemming from the multiple hypotheses screened.

The study has several limitations. Participants were recruited into the study in an ongoing process that spanned over 10 years. All patients of AJ descent were asked to participate resulting in a wide variability of patients' stages and ages. Subjects were screened for the G2019S mutation in the LRRK2 gene and the 7 common mutations in the GBA gene, as those were the known genetic risk factors for PD. It is possible that patients may harbor additional mutations that were not screened at this time, however we expect that in this population their impact would be very low. The protocol was amended several times during this process and thus not all participants had all data points. Data was not imputed but rather all available data for each regression model was used. In our hierarchical procedure, we first selected measures that showed significant evidence, at 0.05 level of FDR control. This eliminated many families of symptoms. However, when performing the same analysis with respect to our selected measures at a higher significance level of 0.1, all selected families yielded a significant association. This implies that an association may exist between the selected families and the genotypes, but the signal is not strong enough to be discovered at the current sample size and at our pre-determined significance level. We included all GBA mutations in the same group due to the small proportion of severe GBA mutations in this cohort and the constraints of the method in use. Based on previous reports, it is possible that the findings may have been driven by the severity of the mutations. This should be further explored in future studies with larger samples from each mutation.

Nevertheless, the uniqueness in this study is the absence of the domain experts in selecting which hypotheses to test, and thus reducing result-bias. By using testing procedures on the whole data set while controlling for the relevant error rate both in the screening stage and in the testing stage, we were able to shed light on multiple genotypes-phenotypes associations at once. The findings from this study provide a more complete portrayal of symptomatic manifestation in genetic PD and could help direct future studies into disease modifying trials and direct personalized treatment approaches.

## Data Availability

Data sharing is unauthorized by our Helsinki committee due to the sensitivity of genetic information and privacy. Anonymized data could be shared on request, as well as the study protocol, statistical plan, and reproducible code.

## Author Contributions

TK, AMit, NG, AMir, and YB: conception and design of the study. TK, AMit, AT, TGu, AO-U, MG-W, NS, TGa, MM-K, KM, SB, NG, AMir, and YB: acquisition and analysis of data. TK, AMit, NG, AMir, and YB: drafting a significant portion of the manuscript or figures.

### Conflict of Interest Statement

SB reports grants from Michael J. Fox Foundation, during the conduct of the study. KM reports grants from NIH UL1TR001873 (Riley), grants from Parkinson's Disease Foundation, and grants from Michael J. Fox, during the conduct of the study; grants from CHDI, grants from HDSA, grants from NIH, grants from TEVA, grants from Vaccinex, and personal fees from Springer LLC, outside the submitted work. NG reports grants from MJF Foundation during the conduct of the study; personal fees from Sanofi, grants and personal fees from Lysosomal Therapeutics, personal fees from Denali, grants and personal fees from Biogen, grants and non-financial support from Michael J. Fox Foundation, outside the submitted work. AM reports grants from Michael J. Fox, and grants from Israeli Science Foundation, during the conduct of the study. The remaining authors declare that the research was conducted in the absence of any commercial or financial relationships that could be construed as a potential conflict of interest.
