# Supplementary material for: Hierarchical Data-Driven Analysis of Clinical Symptoms Among Patients With Parkinson's Disease
Source: Front Neurol. 2019 May 21;10:531. doi: 10.3389/fneur.2019.00531 (PMC6536639; doi:10.3389/fneur.2019.00531)
Supplement: Supplementary file 1 [file Data_Sheet_1.docx]

Supplementary Material

## Supplementary Figures


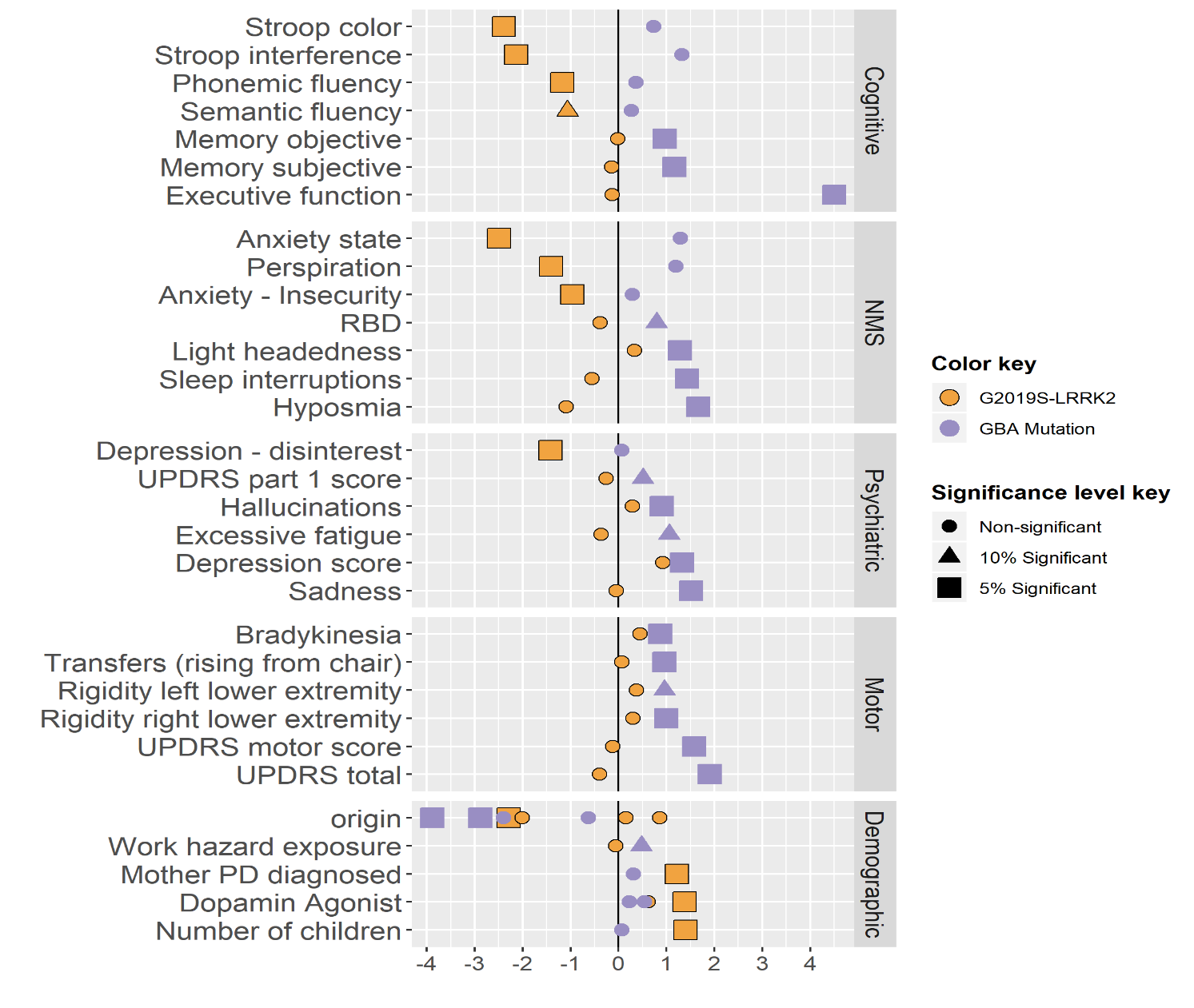


**Supplementary Figure 1.** Standardized effect sizes divided by domains and phenotypes of the two genotypes coefficients. The calculation of the standardized effect sizes varies across the different linear models as follows:

- Linear regression models: $\frac{\beta_{genotype}}{SD\left( y \right)}$
- Logistic regression model (multinomial and binomial models): $\log\left( OR \right)$
- Ordered logistic regression models: $\log\left( Cumulative OR \right)$

The effect sizes are colored by genotype, and their shape and size present the minimal significance level they pass (0.05, 0.1 or none of them).
